# Association of *RANKL* and *OPG* Gene Polymorphism in Arab Women with and without Osteoporosis

**DOI:** 10.3390/genes12020200

**Published:** 2021-01-29

**Authors:** Saba Abdi, Rawan A. Binbaz, Abdul Khader Mohammed, Mohammed G.A. Ansari, Kaiser Wani, Osama E. Amer, Abdullah M. Alnaami, Naji Aljohani, Nasser M. Al-Daghri

**Affiliations:** 1 Biochemistry Department, College of Science, King Saud University, Riyadh 11451, Saudi Arabia; sabdi@ksu.edu.sa (S.A.); rbinbaz@gmail.com (R.A.B.); makhaderonline@gmail.com (A.K.M.); wani.kaiser@gmail.com (K.W.); osamaemam@gmail.com (O.E.A.); aalnaami@yahoo.com (A.M.A.); najij@hotmail.com (N.A.); 2 Sharjah Institute of Medical Research, University of Sharjah, Sharjah 27272, UAE; ansari.bio1@gmail.com; 3 Obesity, Endocrine and Metabolic Center, King Fahad Medical City, Riyadh 59046, Saudi Arabia

**Keywords:** *RANKL*/*OPG*, single nucleotide polymorphism, rs2277438, rs9533156, rs2073618, rs3102735, Saudi postmenopausal women

## Abstract

Receptor activator of the nuclear factor-κB ligand (RANKL) and osteoprotegerin genes (OPG) were identified as susceptible loci for postmenopausal osteoporosis (PMO) in various ethnicities, but neither have been studied in an Arabian population. Hence, the current study aimed to fill this gap. A total of 372 postmenopausal women (174 osteoporosis (OP) and 198 control group (CTRs)) were genotyped for four SNPs: rs2277438A/G and rs9533156T/C (*RANKL*), and rs2073618C/G and rs3102735T/C (*OPG*). Anthropometrics, bone mineral density, 25(OH)D and several other bone markers were measured. The frequency distribution of the heterozygous CG genotype of rs2073618 (*OPG*) was lower in the OP (36.8%) than in CTRs (47%) (OR: 0.6, 95% CI: 0.3–0.97; *p* = 0.041). No differences in the allelic/genotypic frequencies were detected between the two groups for all other studied SNPs. However, the heterozygous TC genotype of rs3102735 (*OPG*) was associated significantly with lower BMD at the femoral neck in OP subjects (*p* = 0.04). The homozygous rare CC genotype of rs9533156 (*RANKL*) was associated with lower 25(OH)D levels in CTRs (*p* = 0.032). In contrast, heterozygous AG genotype of rs2277438 (*RANKL*) is associated with lower 25(OH)D in the OP group (*p* = 0.02). Our results suggest that *RANKL* SNPs may impact 25(OH)D levels and that *OPG* SNP rs2073618A/G is a significant genetic risk factor for PMO Saudi Arabian women.

## 1. Introduction

Osteoporosis is a common skeletal disorder characterized by reduced bone mass and deterioration in bone tissue microarchitecture, predisposing individuals to increased skeletal fracture risk [1,2]. Postmenopausal osteoporosis (PMO) is a global epidemic, and most women remain asymptomatic until a fracture occurs, which eventually results in a heavy financial burden on patients, their families, and society in general [3,4]. Bone mineral density (BMD) is considered one of the best osteoporotic fracture predictors. Although BMD is influenced by several hormonal [5] and environmental factors, data from family and twin studies indicate that 50–85% of BMD variability is genetically determined [6].

Bone remodeling is regulated by the cellular interactions between osteoblasts and osteoclasts. Over the past few decades, receptor activator of nuclear factor-κB (RANK), receptor activator of nuclear factor-κB ligand (RANKL) and osteoprotegerin (OPG) have been emphasized for their role in bone remodeling [7,8]. RANK is expressed by osteoclasts and their precursors and is a member of the tumor necrosis factor family [9]. RANK interacts with its ligand RANKL and regulates bone resorption by controlling the differentiation, proliferation, and overall survival of osteoclasts [10]. OPG, a soluble molecule secreted by osteoblasts, has a strong affinity towards RANKL. Thus, OPG functions as the RANKL decoy receptor and inhibits the interaction of RANKL with RANK, which in turn slows down the bone resorption cycle [11].

Because of the crucial role of OPG and RANKL in bone remodeling, the genes encoding for OPG (TNFRSF11B; gene map locus 8q24.12) and RANKL (TNFSF11; gene map locus 13q14) have been considered as promising candidates for osteoporosis [12,13]. Several studies based on candidate gene association, genome-wide association studies (GWAS), and meta-analyses have highlighted the association of OPG and RANKL gene variants with BMD and osteoporosis risk [14,15,16]. However, most of these studies were done in either East Asian [12,17,18] or European [13,19,20,21] populations or in osteopenia subjects [22]. Hence, more studies in other ethnic populations are warranted to confirm these genes as susceptibility loci. To the best of our knowledge, no such study has been conducted on an Arabian ethnic population. Thus, the present study aimed to assess the potential association of *RANKL* (rs2277438A/G; rs9533156T/C) and *OPG* (rs2073618A/G; rs3102735T/C) gene polymorphisms with the risk of PMO in Saudi Arabian women. In addition, whether SNPs in *RANKL* and *OPG* genes have any influence on BMD and other clinical traits related to bone metabolism will be determined.

## 2. Materials and Methods

### 2.1. Participants

A total of 372 postmenopausal Saudi females (174 osteoporosis (OP) and 198 age-matched healthy controls (CTRs)) aged 50–90 years were enrolled in the study as part of the Osteoporosis Registry of the Chair for Biomarkers of Chronic Diseases (CBCD) in King Saud University, Riyadh, KSA. Participants were recruited consecutively from different primary health care centers (PHCCs) in Riyadh city. Demographic and medical history was obtained using a general questionnaire. Written consent was obtained from all participants prior to inclusion in the registry. 

### 2.2. Exclusion Criteria

Participants with a history of anti-osteoporosis treatment, on medications known to interfere with bone metabolism and with documented bilateral oophorectomy, hypogonadism, hypothyroidism, malignancies, hereditary bone disease or endocrine, cardiac and lung disease as well as those with a creatinine clearance < 30 mL/min were excluded from the study.

### 2.3. Anthropometric and Blood Collection

Blood samples were collected from all participants after an overnight fast (>10 h). Peripheral blood for DNA extraction was collected in EDTA, whereas blood for serum was collected in plain tubes. The serum samples were stored at −20 °C until further analysis. Anthropometric parameters were recorded, including height (rounded off to the nearest 0.5 cm) and weight (rounded off to nearest 0.1 kg), which was measured using an appropriate standard scale (Digital Person Scale; ADAM Equipment, Milford, CT, USA), as well as waist and hip circumference (measured using a standardized tape in cm). Body mass index (BMI) was calculated using the standard equation as kg/m^2^. The systolic and diastolic blood pressure (BP) readings (in mmHg) were taken using appropriate cuffs.

### 2.4. Measurement of Bone Mineral Density (BMD)

For all participants, dual-energy X-ray absorptiometry (DEXA) (Hologic QDR 2000 Inc., Waltham, MA, USA) was used to measure BMD (g/cm^2^). The T-scores recorded were in accordance with World Health Organization guidelines (WHO) (T-score values of −2.5 or less standard deviations (SD) value indicate osteoporosis, T-score values between −1.0 and −2.5 SD indicate osteopenia, and T-score values of −1.0 SD or more as normal). All participants were categorized into two groups (osteoporosis group (OP) and control group (CTRs) based on the presence or absence of osteoporosis.

### 2.5. Biochemical Analysis

Serum OPG levels were measured using the Human Bone Magnetic Bead Panel (Milliplex^®^ Map kit, intra- and inter-assay coefficients of variation (CV): <10% and <15%, respectively). In comparison, other serological parameters like RANKL: intra-assay <10% CV inter-assay <15%, tumor growth factor-β (TGF-β1): intra- and inter-assay CV was <10%, were determined by Human RANKL Magnetic Bead Single Plex Kit and Human TFG-β1 Magnetic Bead Single Plex Kit (Milliplex^®^ Map kit), respectively. Serum 25-hydroxyvitamin D (25(OH)D) was measured by Roche Elecsys modular analytics Cobas e411 using an electrochemiluminescence immunoassay (Roche Diagnostics, GmbH, Mannheim, Germany). Vitamin D binding protein (VDBP) was measured by ELISA (R&D Systems) with inter-assay CV (1.6–3.6%). Serum N-terminal telopeptide (NTx) was determined by ELISA (Alere Scarborough, Inc, Portland, ME, USA), with intra- and interassay CV of 4.6% and 6.9%, respectively. The serum concentration of Sclerostin (SOST), IL-1β, insulin-like growth factor-1 (IGF-1), and parathyroid hormone (PTH) were quantified using the Luminex Multiplex Assay System (LuminexInc, Austin, TX, USA). Intra- and inter-assay coefficients of variation (CV) for IL-1β were 7% and 9%, respectively, whereas for PTH they were 4% and 9%. Osteocalcin (OC) and osteopontin (OPN) were measured using Luminex IS 200 (Luminexcorp).

C-terminal cross-linked telopeptide of type I collagen (CrossLaps) assays were performed with a serum crossLaps ELISA kit (Immunodiagnoztic AG, Bensheim, Germany). Maximum intra- and inter-assay CVs were 3.0% and 10.9%, respectively.

### 2.6. Genetic Analysis

Genomic DNA was extracted from the whole blood using the All Prep DNA mini kit (Qiagen, Hilden, Germany) according to the manufacturer’s instructions. The concentration and purity (260/280) of extracted DNA were checked by a nanodrop ND-1000 spectrophotometer (Thermo Scientific, Gloucester, UK). Four SNPs, two in *RANKL* (rs2277438A/G and rs9533156T/C) and two in the *OPG* gene (rs2073618C/G; rs3102735T/C), were chosen for genotyping. The *RANKL* SNP rs2277438A/G is located at the 5′ untranslated region (UTR). At the same time, rs9533156T/C is found in the promoter region at chromosomal location 13q14. The *OPG* gene SNP rs2073618A/G was located in exon I, whereas rs3102735T/C was found in the promoter region at chromosomal location 8q24. 

All four SNPs were evaluated using allelic discrimination real-time PCR from pre-designed TaqMan genotyping assays (Applied Biosystems, Foster City, CA, USA; rs2277438, assay ID: C__25473654_10; rs9533156, assay ID: C__30009803_10; rs2073618, assay ID: C___1971047_40; rs3102735, assay ID: C___1971046_10) according to protocol mentioned in our previous study [23]. The PCR amplifications were conducted with 30 ng of genomic DNA in a final reaction volume of 10 μL containing 1x TaqMan genotyping Master Mix (Applied Biosystems) and 1x TaqMan genotyping primer assay. All amplifications and detections were performed in 96-well PCR plates using a Bio-Rad CFX96 Real-Time PCR Detection System (Bio-Rad, Milan, Italy). For validation purposes, around forty random samples were re-genotyped and the results were reproducible without discrepancies.

### 2.7. Statistical Analysis

All statistical analyses were conducted using Statistical Package for the Social Sciences for Windows (SPSS version 21.0, IBM, Armonk, NY, USA). Quantitative normal variables and quantitative non-normal variables were expressed as mean ± SD and medians (Q1–Q3). The independent sample t-test and the Mann–Whitney U test were applied for comparisons between groups. Moreover, analysis of variance (ANOVA) and Kruskal–Wallis tests were used to compare different genotypes in each SNP for normal and non-normal variables, respectively. Hardy–Weinberg (H–W) equilibrium tests for association were applied to test if the H–W equilibrium was obeyed. Binary and multivariate binary logistic regression was used to calculate odds ratios (ORs) and 95% confidence intervals of the allele and genotype. The common allele and genotype were used as the reference. Significance was set at *p* < 0.05.

## 3. Results

Participants in the control group were significantly younger (*p* < 0.001) with higher BMI (*p* < 0.001) compared to their counterparts, hence age and BMI were adjusted. Years since menopause were significantly higher (*p* = 0.002) and TGF-β1 levels were significantly lower (*p* = 0.014) in OP subjects than the CTRs. No significant difference was observed between the two studied groups for the remaining anthropometrics and bone markers (Table 1).

The allele and genotype frequency distribution of *RANKL* and *OPG* SNPs are presented in Table 2. Genotype distributions of all SNPs were consistent with the Hardy–Weinberg equilibrium (*p* > 0.05). 

For SNP rs2073618 in *OPG,* a significant adjusted odds ratio of CG genotype (OR = 0.6, 95% CI = 0.3–0.97, *p* = 0.041) and the combination of GG + CG genotypes (OR = 0.6, 95% CI = 0.3–0.9, *p* = 0.029) in OPG rs2073618 indicated that individuals with these genotypes have a 40% lower risk of developing osteoporosis than individuals with the CC genotype. It was also observed that individuals with the G allele (OR = 0.7, 95% CI = 0.5–0.99, *p* = 0.044) also have a 30% lower risk of developing osteoporosis than individuals with the C allele. For the *OPG* SNP rs3102735, the genotypes and alleles occurred at the same frequency between the CTRs and OP and showed no association with the risk of osteoporosis. Similarly, odds ratios indicated that none of the studied *RANKL* gene polymorphisms (rs2277438 A/G, rs9533156 T/C) were associated with osteoporosis risk (Table 2).

Next, we studied the impact of *RANKL* and *OPG* polymorphisms on various clinical traits related to osteoporosis. In the OP group, the heterozygote AG genotype of rs2277438 (*RANKL* gene) was significantly associated with lower serum 25(OH)D levels than the AA genotype in the OP group (*p* = 0.02) (Table 3).

In the CTRs, homo zygote CC genotype of rs9533156 (*RANKL*) was significantly associated with lower 25(OH)D levels than AA genotype (*p* = 0.032) (Table 4).

Furthermore, in the CTRs, the homozygote CC genotype of rs2073618 (*OPG*) was significantly associated with lower total pyridinoline levels than the GG genotype (*p* = 0.005) (Table 5). In the OP group, the heterozygote CT of rs3102735 (*OPG*) was significantly associated with decreased femoral BMD than the TT genotype (*p* = 0.04) (Table 6).

Lastly, the associations of serum concentrations of OPG, RANKL, and TGF-β with anthropometrics of study subjects are summarized in Table 7. In the univariate correlation analysis, OPG correlated positively with age (*r* = 0.3, *p* = 0.01) and inversely with weight (*r*= −0.18, *p* = 0.03).

## 4. Discussion

The direct involvement of OPG and RANKL in bone metabolism makes *OPG* (TNFRSF11B) and RANKL (TNFSF11) essential candidate genes for PMO susceptibility [24]. Polymorphisms in *OPG* and *RANKL* have been associated with BMD or PMO in different genome-wide association studies derived from Europeans and East Asians [25,26]. The pathogenic mechanism of OPG and RANKL gene variants in osteoporosis development remains unclear since previous studies have reported contradictory findings [2,27,28,29]. The present study is the first to provide information on the association of *RANKL* and *OPG* gene polymorphism with osteoporosis phenotype in Saudi Arabian postmenopausal women.

OPG behaves as an inhibitor for osteoclastogenesis by acting as a soluble decoy receptor for RANKL. The SNP rs2073618C/G in the coding region of *OPG* causes the third amino acid lysine to change to asparagine. A similar change has been observed in the signal peptide of angiotensinogen, which has been shown to significantly affect its secretory kinetics [30]. In the current study, we found a significant difference in the allelic and genotypic distribution of *OPG* rs2073618 among participants with and without osteoporosis. Thus, CG heterozygote of *OPG* rs2073618 was significantly associated with reduced risk of PMO. Moreover, the G allele had a protective effect with reference to PMO. This finding is contrary to the study on postmenopausal Scandinavian women, where Langdahl et al. found that women with osteoporosis had a higher frequency of the CG heterozygote for *OPG* SNP rs2073618C/G than those without (56% versus 42.6%) [23]. It has been reported that *OPG* rs2073618C/G is associated with reduced OPG synthesis, which could adversely affect its function as a decoy receptor [31]. However, in the current study, genotype based analysis of OPG rs2073618C/G polymorphisms did not show any difference neither in serum OPG, RANKL concentrations nor BMD. 

We carried out an association analysis to see whether the studied SNPs in *OPG* influenced the BMD, based on the previous models [32]. In postmenopausal women, *OPG* SNP rs3102735 T/C has been associated with lower BMD or higher frequency of fractures related to the minor allele (C) at different bone sites [23,33,34]. These observations support the present study where significantly lower BMD in the left femur was found in the minor allele homozygotes than heterozygotes and major allele homozygotes for *OPG* rs3102735(T > C) in the OP group. Since there is no such correlation found in CTRs group indicates that OPG rs3102735 is not potentially a risk factor for PMO, instead it correlates with BMD at left femur after the osteoporosis diagnosis. 

RANKL is another vital player in osteoporosis outcome, as it has a crucial role in osteoclast differentiation [35]. Human monoclonal anti-RANKL antibody (denosumab) (RANKL inhibitor) has been reported to deter additional bone loss in osteoporosis subjects by inhibiting the development of osteoclasts [36,37]. However, studies focusing on *RANKL* SNPs have yielded varying results. Most of these studies failed to associate rs2277438A/G in the promoter of RANKL with osteoporosis or BMD in postmenopausal women [27,28,37]. In agreement with this, we found no significant association between *RANKL* rs2277438A/G and osteoporosis. Whereas, a study done on postmenopausal Korean women found that genotypes for SNP rs2277438A/G of RANKL combined with the heterozygous genotype for *OPG* SNP rs2073618G/C were significantly associated with BMD at the lumbar spine [38]. Another well-studied polymorphism in the RANKL promoter region is rs9533156C/T. The present study found no significant associations between *RANKL* rs9533156 C/T and osteoporosis or BMD among postmenopausal women. This study, in agreement with several previous studies that did not show any association between RANKL SNP rs9533156 (C > T) and BMD [15,39]. Conversely, an association of *RANKL* rs9533156 (C > T) with lumbar spine BMD and bone loss in the hip and femoral neck has also been reported [40,41]. This inconsistency, too, could be due to ethnic differences.

While we did not find any association of selected *RANKL* SNPs with BMD or PMO in the studied population, it was interesting to note that the CC genotype of rs9533156 (*RANKL*) was associated with lower 25(OH)D levels in CTRs (*p* = 0.032), while the heterozygous AG genotype of rs2277438 (*RANKL*) was associated with lower 25(OH)D in the osteoporosis group (*p* = 0.02). Our results suggest that polymorphism in *RANKL* may play a role in vitamin D status and PMO association. The promoter region of the *RANKL* gene contains vitamin D and glucocorticoid response elements. Earlier studies have reported that the expression of RANKL in cells such as osteoblasts is stimulated by vitamins, and increased RANKL expression enhances bone resorption via an activation of osteoclasts [42].

The current study demonstrated that the value of BMD measured in the CTRs group was comparable to that previously reported in Saudi postmenopausal females [43]. Early menopause is a predisposing factor for osteoporosis [44]. We found that the OP group experienced a significantly extended period of menopause than CTRs. Early estrogen withdrawal results in a rapid decrease in bone mass, disturbed bone architecture, and reduced bone strength compared to those who experience menopause later. The spontaneous increase in pro-inflammatory cytokines following menopause may be involved in this process [45]. It is unclear whether estrogen’s influence on osteoblasts is direct or is linked with various aspects of bone remodeling [46].

The cytokine TGF-β1 is a pleiotropic cytokine, abundant in bone and implicated as an essential regulator of bone remodeling [47]. Several studies attempted to clarify the role of TGF-β1 in the pathogenesis of osteoporosis. These studies indicated that TGF-β1 interacts with hormones and soluble factors, such as estrogen, PTH, and vitamin D. Estrogen has been found to stimulate TGF-β1 production and promote osteoblast proliferation and maturation. Additionally, estrogen also causes osteoclast apoptosis through a TGF-β-dependent pathway [48,49]. The significantly reduced TGFβ1 levels observed in the OP group supports its relevance with increased bone resorption.

It has been proposed previously that estrogen may exert its anti-resorptive effects on bone, at least partially, by stimulating the estrogen receptor and OPG expression in osteoblasts [50]. However, the present study did not find a significant difference in the serum level of OPG among the studied groups. The serum levels of RANKL was also comparable between the two groups. The OPG/RANKL ratio is critical for bone remodeling. However, the expression of OPG and RANKL occurs under the influence of several factors, such as cytokines, glucocorticoid, growth factors, and hormones [51]. The present study found a serum concentration of OPG to correlate directly with age in postmenopausal Saudi women. Some previous studies reported that in postmenopausal women, the serum and bone matrix OPG increases with age, and this is significantly correlated with BMD [31,52,53]; in contrast, others found no association [54]. The rise in OPG with increasing age is considered a compensatory response to enhanced bone resorption by osteoclasts [52]. Studies suggest that body weight (BMI) correlates positively with BMD [55], and serum OPG levels are raised in PMO [56]. This may explain the inverse correlation between body weight and serum OPG levels observed in Saudi postmenopausal women who participated in the present study.

The authors acknowledge several limitations. Participants with osteoporosis were older and experienced a more extended postmenopausal period, which may alter the relation between analyzed polymorphism and osteoporosis. Secondly, we focused only on the common variants in the OPG and RANKL locus. Whether novel polymorphisms or rare mutations within these genes confer risk to BMD remains to be investigated. Nevertheless, the study’s strength includes the homogeneity of the population and the inclusion of most bone markers, providing first-hand evidence on the association of studied SNPs in the Arabian ethnic group of postmenopausal women.

## 5. Conclusions

The results of this study found no association of *RANKL* polymorphism with osteoporosis in postmenopausal Arab women. However, *RANKL* SNPs may impact 25(OH)D levels in this population. The distribution of genotypes for *OPG* SNP rs2073618 (G > C) showed a significant association with osteoporosis, suggesting a role in the genetic susceptibility to osteoporosis among postmenopausal Arab women. Further studies are required to clearly define the association of rs2073618 (G > C) genetic variant or other SNPs in *OPG* with BMD and osteoporosis to understand the disease pathogenesis better. 

## Figures and Tables

**Table 1:** Anthropometric, metabolic and bone markers of participants according to groups.

Parameter	OP	CTRs	*p*-Value	*p*-Value *
*N*	174	198		
Age (years)	58.7 ± 7.8	53.4 ± 5.9	<0.001	-
BMI (kg/m^2^)	30.3 ± 6.1	34.3 ± 5.3	<0.001	-
WC (cm)	96.4 ± 14.9	102.5 ± 13.2	<0.001	0.83
HC (cm)	106.3 ± 13.5	112.6 ± 12.5	<0.001	0.57
WHR	0.9 ± 0.1	0.9 ± 0.1	0.49	0.41
Age of Menarche (Years)	13.3 ± 1.6	13.1 ± 1.4	0.18	0.93
Menopause (Years) ^#^	10.0 (4.0–15.0)	4.0 (2.0–7.0)	<0.001	0.002
T- score AP Spine L1-L4 ^#^	−2.9 (−3.4–−2.7)	−0.3 (−0.7–0.3)	<0.001	<0.001
T-Score (DF-left) ^#^	−1.4 (−2.0–−0.9)	0.7 (0.1–1.3)	<0.001	<0.001
BMD (Spine)	0.82 ± 0.06	1.2 ± 0.1	<0.001	<0.001
BMD (DF-Left)	0.80 ± 0.13	1.1 ± 0.1	<0.001	<0.001
BMD (DF-Right)	0.81 ± 0.13	1.1 ± 0.1	<0.001	<0.001
OPG (pg/mL) ^#^	0.9 (0.7–1.3)	0.7 (0.5–0.9)	0.001	0.15
RANKL (pg/mL) ^#^	30.9 (22.1–54.7)	34.6 (20.9–65.5)	0.46	0.13
RANKL/OPG ^#^	0.03 (0.01–0.05)	0.04 (0.03–0.06)	0.10	0.31
25(OH)D (nmol/L)	73.1 (39.1–100.1)	58.0 (34.4–82.3)	0.06	0.58
VDBP (µg/mL)	11.3 (6.0–70.1)	11.0 (5.9–46.6)	0.41	0.19
PTH (pg/mL)	15.7 (7.2–29.0)	11.5 (6.7–20.4)	0.08	0.30
OC (ng/mL)	12.7 (6.1–19.5)	6.8 (3.3–12.9)	0.025	0.19
OPN (ng/mL)	2.6 (1.3–3.9)	2.3 (1.4–3.5)	0.78	0.25
SOST (ng/mL)	1.5 (0.9–2.2)	1.5 (0.7–2.7)	0.95	0.53
NTx (nmol/L)	56.1 (40.8–77.2)	49.1 (38.1–62.6)	0.32	0.63
β-crosslap (ng/mL)	0.1 (0.0–0.1)	0.1 (0.1–0.1)	0.72	0.75
T. Pyrid (ng/mL)	12.3 (7.3–20.7)	15.3 (7.0–31.3)	0.23	0.07
TGF-β (ng/mL)	35.0 (18.1–47.8)	41.5 (35.2–53.0)	0.003	0.014
IGF-1 (ng/mL)	18.4 (13.3–40.3)	17.7 (11.9–43.0)	0.80	0.62
IL-1β (pg/mL)	1.7 (0.4–2.7)	1.6 (0.4–2.7)	0.59	0.87

**Note**: Data presented as mean ± standard deviation for normal variables while the median (quartile 1–quartile 3) presented for non-normal variables; ^#^ indicates non-normal variables; *p*-value < 0.05 considered significant, * indicates adjusted *p*-value for age and BMI; body mass index (BMI), waist circumference (WC), hip circumference (HC), hip to waist ratio (WHR), bone mineral density (BMD), dual femur (DF), osteoprotegerin (OPG), receptor activator of the nuclear factor-κB ligand (RANKL), serum 25-hydroxy vitamin D (25 (OH)D), vitamin D binding protein (VDBP), parathyroid hormone (PTH), osteocalcin (OC), osteopontin (OPN), the serum concentration of sclerostin (SOST), serum N-terminal telopeptide (NTx), C-terminal cross-linked telopeptide of type I collagen (β-cross lap), total pyridinoline (T.Pyrid), tumor growth factor β (TGF-β), insulin-like growth factor-1(IGF-1), interleukin-1β (IL-1β).

**Table 2:** Association between receptor activator of the nuclear factor-κB ligand (*RANKL*) and osteoprotegerin (*OPG*) gene polymorphisms and osteoporosis risk.

	OP *N* (%)	CTRs *N* (%)	OR (95% CI)	*p*-Value	Adjusted OR	*p*-Value *
*RANKL* gene polymorphism						
rs2277438 A/G						
AA	122 (73.1)	140 (74.5)	1.0		1.0	
AG	42 (25.1)	45 (23.9)	1.0 (0.6–1.6)	0.96	0.9 (0.5–1.6)	0.83
GG	3 (1.8)	3 (1.6)	1.1 (0.2–5.7)	0.88	2.7 (0.4–20.2)	0.32
AG + GG	45 (26.9)	48 (25.5)	1.0 (0.6–1.6)	0.98	1.0 (0.6–1.7)	1.00
A	286 (85.6)	325 (86.4)	1.0		1.0	
G	48 (14.4)	51 (13.6)	1.0 (0.7–1.5)	0.98	1.1 (0.6–1.7)	0.81
rs9533156 T/C						
TT	73 (42.0)	85 (42.9)	1.0		1.0	
TC	76 (43.7)	75 (37.9)	1.2 (0.8–1.8)	0.42	1.2 (0.8–1.9)	0.42
CC	25 (14.4)	38 (19.2)	0.8 (0.4–1.4)	0.41	0.7 (0.4–1.3)	0.28
TC + CC	101 (48.0)	113 (57.1)	1.1 (0.7–1.6)	0.79	1.0 (0.7–1.6)	0.87
T	222 (63.8)	245 (61.9)	1.0		1.0	
C	126 (36.2)	151 (38.1)	0.9 (0.7–1.2)	0.63	0.9 (0.6–1.2)	0.48
*OPG* gene polymorphism						
rs2073618 C/G						
CC	87 (50.0)	85 (42.9)	1.0		1.0	
CG	64 (36.8)	93 (47)	0.7 (0.4–1.0)	0.08	0.6 (0.3–0.97)	0.041
GG	23 (13.2)	20 (10.1)	1.1 (0.6–2.2)	0.75	0.6 (0.2–1.3)	0.17
GG + CG	857 (50.0)	113 (57.1)	0.8 (0.5–1.1)	0.17	0.6 (0.3–0.9)	0.029
C	238 (68.4)	263 (66.4)	1.0		1.0	
G	110 (31.6)	133 (33.6)	0.9 (0.7–1.2)	0.57	0.7 (0.5–0.99)	0.044
rs3102735 T/C						
TT	113 (79.0)	127 (77.4)	1.0		1.0	
TC	27 (18.9)	36 (22.0)	0.8 (0.5–1.4)	0.49	0.7 (0.3–1.3)	0.20
CC	3 (2.1)	1 (0.6)	3.4 (0.4–32.9)	0.29	1.6 (0.1–24.5)	0.72
TC + CC	30 (21.0)	37 (22.6)	0.9 (0.5–1.5)	0.67	0.7 (0.4–1.3)	0.25
T	253 (88.5)	290 (88.4)	1.0		1.0	
C	33 (11.5)	38 (11.6)	1.0 (0.6–1.6)	0.92	0.8 (0.4–1.4)	0.34

**Note**: Data presented as OR (95% CI), * indicates adjusted for age and BMI and menopause (years). *p*-value < 0.05 considered significant.

**Table 3:** Clinical characterization according to rs2277438 A/G (*RANKL*) in the control and osteoporosis group.

Parameters	OP				CTRs			
rs2277438 A/G (*RANKL*)	GG (*N* = 3)	AG (*N* = 42)	AA (*N* = 73)	*p*-Value	GG (*N* = 3)	AG (*N* = 45)	AA (*N* = 140)	*p*-Value
Menopause (years)	13.0 (10.0–15.0)	10.0 (5.0–15.0)	8.0 (4.0–15.0)	0.19	4.5 (4.0–5.0)	4.0 (3.0–7.0)	4.0 (2.0–7.0)	0.26
BMD Spine	0.70 ± 0.00	0.81 ± 0.06	0.83 ± 0.05	0.18	1.2 ± 0.0	1.2 ± 0.1	1.16 ± 0.12	0.16
BMD (DF left)	0.73 ± 0.15	0.82 ± 0.15	0.80 ± 0.12	0.41	1.1 ± 0.1	1.03 ± 0.12	1.05 ± 0.12	0.30
BMD (DF right)	0.77 ± 0.15	0.81 ± 0.14	0.81 ± 0.12	0.88	1.0 ± 0.1	1.04 ± 0.12	1.04 ± 0.12	0.78
Average BMD	0.73 ± 0.17	0.82 ± 0.13	0.81 ± 0.11	0.70	1.0 ± 0.1	1.05 ± 0.12	1.06 ± 0.11	0.80
25(OH) D (nmol/L)	70 (68–72)	62.2 (28.3–84.4)	80.7 (41.3–102.4)	0.02	68 (45–81)	53 (29–78)	57.8 (34.4–82.9)	0.66
VDBP (µg/mL)	5.4 (5.4–5.4)	7.0 (5.9–29.3)	20.2 (6.5–85.4)	0.15	17.1 (6.0–28.2)	6.4 (5.4–11.2)	19.0 (6.2–53.8)	0.10
PTH (pg/mL)	0	16.8 (7.6–41.2)	11.4 (7.0–43.5)	0.58	13.2 (2.2–24.2)	8.0 (5.5–12.5)	12.2 (7.1–20.4)	0.67
OPG (ng/mL)	0.8 (0.8–0.8)	0.8 (0.7–1.4)	1.0 (0.7–1.2)	0.93	1.0 (0.8–1.1)	0.8 (0.7–1.1)	0.6 (0.5–0.9)	0.07
RANK-L (ng/mL)	16.6 (5.4–27.7)	34.4(23.0–54.7)	32.1(20.4–54.9)	0.45	65.1 (65.1–65.1)	35.2 (25.3–64.5)	33.7 (20.9–66.1)	0.77
OC (ng/mL)	10.4 (10.4–10.4)	14.8 (8.2–22.4)	10.8 (5.7–17.0)	0.41	25.9 (20.4–31.3)	8.1 (3.8–16.2)	6.7 (3.3–12.2)	0.29
OPN (ng/mL)	3.1 (3.1–3.1)	3.2 (2.6–4.4)	2.3 (1.2–3.7)	0.07	4.8 (1.9–7.7)	2.3 (1.8–3.0)	2.4 (1.2–3.6)	0.87
SOST (ng/mL)	2.0 (2.0–2.0)	1.4 (1.1–2.5)	1.6 (0.9–2.3)	0.88	2.1 (0.9–3.2)	1.1 (0.6–2.2)	1.5 (0.8–2.8)	0.23
NTx (nmol/L)	65.7 (31.9–99.4)	55.3 (43.0–92.9)	58.4 (40.9–68.3)	0.49	44.4 (44.4–44.4)	49.6 (33.4–68.2)	50.4 (41.4–62.6)	0.37
β-crosslap (ng/mL)	0	0.1 (0.0–0.1)	0.1 (0.1–0.2)	0.23	0.1 (0.1–0.1)	0.0 (0.0–0.2)	0.1 (0.1–0.1)	0.96
T. Pyrid (ng/mL)	0	12.3 (9.2–20.7)	11.7 (6.0–21.9)	0.66	8.3 (8.3–8.3)	15.5 (7.5–36.0)	18.8 (5.4–33.7)	0.63
TGF-β (ng/mL)	17.9 (17.9–17.9)	41.2 (18.9–52.0)	34.5 (20.6–47.8)	0.97	47.5 (47.5–47.5)	38.9 (34.0–42.9)	42.1 (35.6–53.4)	0.59
IGF-1 (ng/mL)	0	18.2 (14.2–40.3)	19.1 (13.3–38.2)	0.94	11.3 (11.3–11.3)	13.3 (11.9–38.4)	18.3 (12.1–43.0)	0.88
IL1β (pg/mL)	5.7 (5.7–5.7)	1.7 (0.4–2.7)	1.6 (0.3–2.8)	0.86	0.4 (0.30.4)	1.6 (0.6–2.3)	1.7 (0.3–2.7)	0.68

**Note**: Data presented as mean ± standard deviation for normal variables and median (1st quartile–3rd quartile) for non-normal variables; *p*-value < 0.05 considered significant; GG genotype was excluded from ANOVA analysis because of limited sample size. Body mass index (BMI), waist circumference (WC), hip circumference (HC), hip to waist ratio (WHR), bone mineral density (BMD), dual femur (DF), osteoprotegerin genes (OPG), receptor activator of the nuclear factor-κB ligand (RANKL), serum 25-hydroxy vitamin D (25 (OH)D), vitamin D binding protein (VDBP), parathyroid hormone (PTH), osteocalcin (OC), osteopontin (OPN), the serum concentration of sclerostin (SOST), serum N-terminal telopeptide (NTx), total pyridinoline (T.Pyrid), C-terminal cross-linked telopeptide of type I collagen (β-cross lap), tumor growth factor β (TGF-β), insulin-like growth factor-1(IGF-1), interleukin-1β (IL-1β).

**Table 4:** Clinical characterization according to rs9533156 T/C (*RANKL*) in the control and osteoporosis group.

Parameters	OP				CTRs			
rs9533156 T/C (*RANKL*)	TT (*N* = 73)	TC (*N* = 76)	CC (*N* = 25)	*p*-Value	TT (*N* = 85)	TC (*N* = 75)	CC (*N* = 38)	*p*-Value
Menopause (years)	13.0 (10.0–15)	10.0 (5.0–15.0)	8.0 (4.0–15.0)	0.83	4.0 (2.0–7.0)	5.0 (3.0–7.0)	4.0 (2.0–5.0)	0.15
BMD Spine	0.80 ± 0.07	0.83 ± 0.05	0.82 ± 0.05	0.19	1.16 ± 0.12	1.17 ± 0.08	1.18 ± 0.16	0.82
BMD (DF Left)	0.80 ± 0.14	0.81 ± 0.12	0.78 ± 0.09	0.58	1.05 ± 0.11	1.04 ± 0.13	1.05 ± 0.13	0.91
BMD (DF right)	0.81 ± 0.15	0.81 ± 0.12	0.80 ± 0.08	0.85	1.05 ± 0.11	1.03 ± 0.10	1.05 ± 0.15	0.55
Average BMD	0.81 ± 0.14	0.82 ± 0.12	0.79 ± 0.08	0.71	1.06 ± 0.11	1.03 ± 0.10	1.05 ± 0.15	0.66
25(OH)D (nmol/L)	71.6 (36.7–100)	78.1 (40.2–99.5)	74.7 (51.7–102.4)	0.25	65.5 (43.8–83.9)	54.1 (31.7–80.9)	44.4 (25–65.2) ^A^	0.032
VDBP (µg/mL)	23.4 (6.0–142.5)	9.7 (5.9–50.9)	24.9 (11.8–42.1)	0.44	15.5 (6.1–53.4)	11.5 (5.9–32.2)	5.9 (3.9–19.3)	0.41
PTH (pg/mL)	11.8 (7.9–55.7)	16.8 (7.0–20.0)	18.4 (7.6–48.1)	0.84	10.5 (6.4–22.1)	11.6 (6.7–20.6)	13.8 (6.7–20.4)	0.74
OPG (ng/mL)	0.8 (0.6–1.0)	0.9 (0.7–1.5)	1.0 (1.0–1.5)	0.06	0.7 (0.5–1.0)	0.8 (0.6–0.9)	0.6 (0.5–1.1)	0.88
RANK-L (ng/mL)	29.9 (21.4–54.9)	37.7(24.7–54.7)	28.3 (12.7–46.7)	0.52	31.4 (20.9–65.1)	39.5 (21.2–65.5)	44.4 (20.9–69.3)	0.99
OC (ng/mL)	9.9 (4.9–15.6)	15.1 (5.7–22.4)	14.3 (9.9–21.0)	0.07	7.0 (2.6–15.9)	6.7 (5.5–12.9)	4.6 (3.3–10.2)	0.55
OPN (ng/mL)	2.7 (1.3–4.1)	2.5 (1.2–3.6)	2.8 (1.5–3.9)	0.92	2.3 (1.2–3.5)	2.3 (1.5–3.5)	2.3 (0.8–3.9)	0.70
SOST (ng/mL)	1.4 (0.9–2.2)	1.5 (0.7–2.1)	2.4 (1.5–3.2)	0.31	1.5 (0.7–2.9)	2.0 (0.7–2.6)	1.2 (0.7–2.6)	0.87
NTx (nmol/L)	54.7 (40.8–64.5)	60.6 (33.7–86.4)	52.4 (43.5–62.9)	0.91	47.5 (42.3–58.0)	48.8 (38.1–62.6)	52.2 (35.6–78.6)	0.61
β-crosslap (ng/mL)	0.1 (0.0–0.1)	0.1 (0.1–0.2)	0.1 (0.1–0.2)	0.14	0.1 (0.0–0.1)	0.1 (0.1–0.2)	0.1 (0.0–0.2)	0.22
T. Pyrid (ng/mL)	8.4 (3.0–12.7)	17.1 (10.8–20.7)	15.0 (7.8–33.5)	0.09	16.2 (6.4–28.6)	15.4 (7.4–34.4)	12.3 (2.7–33.7)	0.46
TGF-β (ng/mL)	35.0 (17.9–43.9)	37.5 (18.9–53.3)	33.9 (8.2–50.8)	0.56	41.2 (34.0–53.4)	40.2 (35.1–53.2)	43.5 (42.0–46.1)	0.86
IGF-1 (ng/mL)	15.0 (13.5–44.0)	19.7 (15.6–38.6)	17.2 (6.1–38.2)	0.60	18.0 (10.4–43.5)	19.3 (13.0–47.4)	12.6 (12.6–15.4)	0.44
IL1β (pg/mL)	1.6 (0.4–2.7)	1.8 (0.3–2.8)	1.8 (0.3–2.1)	0.83	1.6 (0.3–2.7)	1.6 (0.4–2.5)	1.8 (1.6–2.9)	0.22

**Note**: Data presented as mean ± standard deviation for normal variables and median (1st quartile–3rd quartile) for non-normal variables; *p*-value < 0.05 considered significant; superscript A indicates significance from TT. Body mass index (BMI), waist circumference (WC), hip circumference (HC), hip to waist ratio (WHR), bone mineral density (BMD), dual femur (DF), osteoprotegerin genes (OPG), receptor activator of the nuclear factor-κB ligand (RANKL), serum 25-hydroxy vitamin D (25 (OH)D), vitamin D binding protein (VDBP), parathyroid hormone (PTH), osteocalcin (OC), osteopontin (OPN), the serum concentration of sclerostin (SOST), serum N-terminal telopeptide (NTx), total pyridinoline (T.Pyrid), C-terminal cross-linked telopeptide of type I collagen (β-cross lap), tumor growth factor β (TGF-β), insulin-like growth factor-1(IGF-1), interleukin-1β (IL-1β).

**Table 5:** Clinical characterization according to rs2073618 C/G (OPG) in the control and osteoporosis group.

Parameters	OP				CTRs			
rs2073618 C/G (*OPG*)	GG (*n* = 23)	CG (*n* = 4)	CC (*n* = 87)	*p*-Value	GG (*n* = 20)	CG (*n* = 93)	CC (*n* = 85)	*p*-Value
Menopause (years)	13.0 (10–15.0)	10.0 (5.0–15.0)	12.0 (4.0–15.0)	0.74	6.5 (4.0–10.0)	4.0 (2.0–7.0)	4.0 (2.0–5.0)	0.09
BMD Spine	0.79 ± 0.06	0.82 ± 0.06	0.82 ± 0.05	0.18	1.20 ± 0.11	1.16 ± 0.09	1.17 ± 0.13	0.54
BMD (DF left)	0.76 ± 0.11	0.80 ± 0.11	0.81 ± 0.14	0.26	1.10 ± 0.11	1.03 ± 0.11	1.05 ± 0.14	0.10
BMD (DF right)	0.78 ± 0.11	0.80 ± 0.12	0.83 ± 0.14	0.20	1.08 ± 0.11	1.03 ± 0.11	1.04 ± 0.13	0.16
Average BMD	0.78 ± 0.11	0.81 ± 0.11	0.82 ± 0.13	0.23	1.09 ± 0.10	1.04 ± 0.10	1.06 ± 0.12	0.15
25(OH)D (nmol/L)	88.6 (42–100)	70.5 (46–106.4)	73.5 (38.2–99.9)	0.67	60.7 (37.6–71.6)	65.2 (42.4–89.5)	47.4 (31.7–74.2)	0.06
VDBP (µg/mL)	37.5 (24.9–69.9)	8.5 (5.9–70.1)	10.7 (6.0–100.1)	0.36	19.6 (6.7–43.6)	10.8 (6.1–33.9)	9.9 (5.6–79.4)	0.91
PTH (pg/mL)	18.0 (7.9–128.3)	10.6 (5.9–18.1)	22.3 (9.3–51.9)	0.07	12.5 (10.0–19.6)	8.4 (5.4–15.7)	13.8 (8.8–32.0)	0.08
OPG (ng/mL)	1.0 (0.6–1.2)	0.9 (0.7–1.5)	1.0 (0.7–1.3)	0.77	0.7 (0.6–1.0)	0.8 (0.5–1.0)	0.6 (0.5–0.9)	0.30
RANK-L (ng/mL)	29.0 (10.5–65.3)	35.0 (24.8–49.7)	31.5 (22.1–54.7)	0.60	29.8 (24.0–78.0)	40.7 (18.4–64.0)	32.0 (21.2–67.3)	0.74
OC (ng/mL)	11.8 (6.1–15.3)	10.4 (4.6–16.8)	14.3 (7.6–22.9)	0.39	9.9 (3.5–17.2)	6.4 (2.6–11.9)	6.8 (4.1–12.9)	0.59
OPN (ng/mL)	3.2 (1.7–4.5)	2.6 (1.2–3.2)	2.7 (1.3–4.1)	0.52	2.7 (1.8–6.4)	2.3 (1.4–3.3)	2.1 (1.2–3.5)	0.56
SOST (ng/mL)	2.0 (1.6–3.6)	1.4 (0.9–2.1)	1.5 (0.9–2.2)	0.18	2.3 (1.0–3.2)	1.3 (0.4–2.9)	1.5 (0.7–2.3)	0.54
NTx (nmol/L)	37.4 (27.8–48.8)	58.7 (34.9–83.4)	59.2 (50.1–72.7)	0.06	41.3 (32.2–51.8)	53.3 (35.2–66.3)	46.1 (41.4–62.6)	0.50
β-crosslap (ng/mL)	0.0 (0.0–0.1)	0.1 (0.1–0.2)	0.1 (0.1–0.1)	0.41	0.1 (0.1–0.1)	0.1 (0.0–0.1)	0.1 (0.1–0.1)	0.58
T. Pyrid (ng/mL)	13.2 (11.2–16.9)	10.3 (7.6–19.6)	12.4 (5.5–24.7)	0.94	35.2 (25.3–56.5)	26.5 (7.9–34.4)	10.3 (5.0–19.5) *A*	0.005
TGF-β (ng/mL)	23.4 (13.5–43.8)	34.0 (18.9–46.5)	38.0 (17.5–50.5)	0.76	43.2 (29.4–63.1)	42.9 (34.0–52.8)	40.7 (35.4–52.2)	0.91
IGF-1 (ng/mL)	38.2 (13.5–55.1)	20.0 (12.0–58.5)	16.5 (13.0–21.8)	0.22	21.0 (17.0–23.2)	17.8 (10.4–64.6)	14.8 (11.3–38.4)	0.90
IL1β (pg/mL)	1.6 (0.2–2.9)	1.6 (0.4–2.2)	1.9 (0.4–2.9)	0.52	1.7 (0.3–2.8)	1.6 (0.3–1.9)	1.7 (0.4–2.8)	0.42

**Note:** Data presented as mean ± standard deviation for normal variables and median (1st quartile–3rd quartile) for non-normal variables; *p*-value < 0.05 considered significant; superscript A indicates significance from GG. Body mass index (BMI), waist circumference (WC), hip circumference (HC), hip to waist ratio (WHR), bone mineral density (BMD), dual femur (DF), osteoprotegerin genes (OPG), receptor activator of the nuclear factor-κB ligand (*RANKL*), serum 25-hydroxy vitamin D (25 (OH)D), vitamin D binding protein (VDBP), parathyroid hormone (PTH), osteocalcin (OC), Osteopontin (OPN), the serum concentration of sclerostin (SOST), serum N-terminal telopeptide (NTx), total pyridinoline (T.Pyrid), C-terminal cross-linked telopeptide of type I collagen (β-cross lap), tumor growth factor β (TGF-β), insulin-like growth factor-1(IGF-1), interleukin-1β (IL-1β).

**Table 6:** Clinical characterization according to rs3102735 T/C (*OPG*) in the control and osteoporosis group.

Parameters	OP				CTRs			
rs3102735 T/C (*OPG*)	TT (*n* = 113)	TC (*n* = 27)	CC (*n* = 3)	*p*-Value	TT (*n* = 127)	TC (*n* = 36)	CC (*n* = 1)	*p*-Value
Menopause (years)	13.0 (10.0–15.0)	11.0 (5.0–15.0)	12.0 (4.0–15.0)	0.52	4.0 (2.0–8.0)	4.0 (2.0–6.5)	7.0 (7.0–7.0)	0.43
BMD Spine	0.82 ± 0.06	0.83 ± 0.04	0.84 ± 0.06	0.68	1.18 ± 0.10	1.14 ± 0.18	1.19 ± 0.00	0.26
BMD (DF left)	0.82 ± 0.13	0.76 ± 0.12 ^A^	0.74 ± 0.17	0.04	1.05 ± 0.12	1.04 ± 0.13	1.30 ± 0.00	0.87
BMD (DF right)	0.82 ± 0.13	0.79 ± 0.12	0.80 ± 0.19	0.64	1.04 ± 0.11	1.05 ± 0.14	1.20 ± 0.00	0.57
Average BMD	0.82 ± 0.12	0.79 ± 0.12	0.79 ± 0.18	0.14	1.05 ± 0.11	1.06 ± 0.13	1.24 ± 0.00	0.61
25(OH) D (nmol/L)	79.9 (45.7–112.7)	64.3 (38.3–96.3)	-	0.06	59.2 (38.8–83.8)	61.0 (33.7–80.5)	90.9 (90.9–90.9)	0.52
VDBP (µg/mL)	20.2 (6.5–100.1)	60.5 (30.8–129.7)	-	0.30	15.5 (4.1–53.8)	14.7 (5.2–32.1)	120.4 (120–120)	0.58
PTH (pg/mL)	17.4 (9.3–26.3)	11.3 (2.8–15.7)	-	0.10	12.2 (7.6–20.4)	9.7 (4.4–15.0)	26.7 (26.7–26.7)	0.08
OPG (ng/mL)	0.8 (0.6–1.1)	1.5 (1.0–1.8) ^A^	-	0.003	0.7 (0.5–0.8)	0.7 (0.4–1.3)	0.6 (0.6–0.6)	0.57
RANK-L (ng/mL)	32.4 (21.5–52.6)	44.1 (27.5–71.0)	51.8 (15.6–88.0)	0.19	34.3 (21.2–64.8)	19.4 (11.3–67.0)	18.4 (18.4–18.4)	0.12
OC (ng/mL)	10.5 (5.7–21.0)	15.3 (4.4–26.7)	-	0.62	6.3 (2.6–12.8)	3.0 (1.8–6.9)	4.6 (4.6–4.6)	0.19
OPN (ng/mL)	3.0 (1.3–3.9)	2.7 (2.1–3.8)	-	0.90	2.3 (1.2–3.6)	1.5 (0.8–2.8)	5.0 (5.0–5.0)	0.15
SOST (ng/mL)	1.4 (0.9–2.5)	1.8 (0.9–2.3)	-	0.96	1.5 (0.7–2.8)	1.3 (0.3–2.9)	4.9 (4.9–4.9)	0.26
NTx (nmol/L)	51.0 (33.7–77.0)	72.2 (49.2–85.2)	-	0.39	52.3 (37.1–57.8)	47.2 (39.3–129.7)	-	0.19
β-crosslap (ng/mL)	0.1 (0.1–0.1)	0.1 (0.0–0.2)	-	0.61	0.1 (0.1–0.1)	0.1 (0.0–0.2)	-	0.67
T. Pyrid (ng/mL)	15.0 (8.7–22.4)	10.6 (3.4–18.1)	-	0.19	14.9 (4.8–28.4)	12.4 (9.3–21.3)	-	0.96
TGF-β (ng/mL)	43.8 (32.3–50.8)	32.1 (13.8–35.7)	-	0.11	41.6 (31.9–52.5)	53.4 (51.6–53.4)	-	0.14
IGF-1 (ng/mL)	19.1 (14.3–44.0)	29.6 (14.4–83.7)	-	0.59	22.4 (15.0–50.8)	18.2 (11.9–43.0)	12.6 (12.6–12.6)	0.57
IL-1β (pg/mL)	1.8 (0.5–2.5)	1.6 (0.4–2.4)	-	0.74	1.6 (0.3–2.1)	1.7 (0.9–2.4)	2.1 (2.1–2.1)	0.70

**Note:** Data presented as mean ± standard deviation for normal variables and median (1st quartile–3rd quartile) for non-normal variables; *p*-value < 0.05 considered significant; CC genotype was excluded from analysis because of limited sample size; superscript A indicates significance from TT. Body mass index (BMI), waist circumference (WC), hip circumference (HC), hip to waist ratio (WHR), bone mineral density (BMD), dual femur (DF),, osteoprotegerin genes (OPG), receptor \activator of the nuclear factor-κB ligand (RANKL), serum 25-hydroxy vitamin D (25 (OH)D), vitamin D binding protein (VDBP), parathyroid hormone (PTH), osteocalcin (OC), osteopontin (OPN), the serum concentration of sclerostin (SOST), serum N-terminal telopeptide (NTx), C-terminal cross-linked telopeptide of type I collagen (β-cross lap), tumor growth factor β (TGF-β), total pyridinoline (T.Pyrid), insulin-like growth factor-1(IGF-1), interleukin-1β (IL-1β).

**Table 7:** Associations of select bone markers with anthropometrics.

Parameter	OPG (ng/mL)		RANKL (pg/mL)		TGF- β (ng/mL)	
	*R*	*p*-Value	*R*	*p*-Value	*R*	*p*-Value
Age (years)	0.30	0.01	0.02	0.81	−0.10	0.30
BMI (kg/m^2^)	−0.16	0.06	−0.02	0.73	0.13	0.19
Weight (Kg)	−0.18	0.03	−0.05	0.52	0.13	0.20
WHR	−0.14	0.11	0.01	0.84	0.10	0.33

**Note:** R indicates a Spearman correlation coefficient; *p* < 0.05 considered significant. Body mass index (BMI), hip to waist ratio (WHR).

## Data Availability

The data presented in this study are available on request from the corresponding author.
